# Interleukin-1 Genotype in Periodontitis

**DOI:** 10.1007/s00005-019-00555-4

**Published:** 2019-07-19

**Authors:** Aniela Brodzikowska, Renata Górska, Jan Kowalski

**Affiliations:** 1 Department of Conservative Dentistry, Medical University of Warsaw, Warsaw, Poland; 2 Department of Periodontology, Medical University of Warsaw, Warsaw, Poland

**Keywords:** Periodontitis, Genotype, IL-1, Polymorphisms

## Abstract

This paper presents the current knowledge concerning the role of polymorphisms of IL1A and IL1B genes in periodontitis. Attention has been paid to the role of IL-1 in the pathogenesis of the disease, and to the significance of a genetic test, investigating the presence of composite two polymorphisms of IL-1 gene, as a risk factor for severe periodontitis. The significance of this test for prevention of periodontitis and its therapy has been discussed. IL-1 polymorphisms have been presented and described according to the reference single nucleotide polymorphism (SNP) identification number (rsID), established to eradicate the redundancy of reported polymorphisms in the SNP database processed by the National Center for Biotechnology Information. The prevalence of these genotypes in different populations and ethnic groups and its effect on periodontal health have been discussed. The presented data show inconsistent results. It seems that at least two polymorphisms, rs1800587 and rs1143634, are associated with periodontal inflammation. Therefore, they can be regarded as candidate genes involved in further periodontitis risk assessment. It seems that geographical and ethnical factors can play a great role, as the prevalence of specific polymorphisms varies greatly depending on the population studied.

## Introduction

Periodontitis is caused by pathogenic microorganisms that activate an immunoinflammatory response of the host. Mediators released by inflammatory cells stimulate periodontal cells to secrete metalloproteinases, proteolytic enzymes directly responsible for connective tissue destruction and prostaglandins, contributing to the destruction of the alveolar bone (van Dyke and van Winkelhoff 2013). Previous studies showed significant individual differences in susceptibility to periodontitis and in the course of the disease. One of the factors contributing to individual differences may be genetic determinants (Ronderos and Ryder 2004). Particular attention was paid to the genetic factors of the immunoinflammatory response, including the role of polymorphisms of genes encoding the production of inflammatory mediators (Schenkein 2002).

Gene polymorphisms encoding interleukin (IL)-1 are the most prominent gene polymorphisms in studies on periodontitis (Greenstein and Hart 2002). IL-1 is a family of at least ten molecules, of which the two most significant ones in the pathogenesis of periodontitis are IL-1α, connected with the cell, and IL-1β, released into the environment and showing agonistic action upon binding with receptor (Boch et al. 2001).

IL-1 participates in a number of processes necessary to initiate and sustain an inflammatory response. It increases the production of adhesion molecules, facilitating leukocyte migration, stimulates the production of other inflammatory mediators and metalloproteinases, activates T and B lymphocytes, stimulates osteoblasts leading to bone resorption, and stimulates programmed death of cells producing extracellular matrix, thus limiting regenerative capabilities of tissues (Graves and Cochran 2003).

IL-1α and IL-1β are encoded, respectively, by IL1A, IL1B genes, located near each other on the long arm (q arm) of chromosome 2 and having common DNA sequences (Greenstein and Hart 2002). Each of these genes is polymorphous (Cox et al. 1998). Because differences in the amount or function of IL-1 produced in response to a bacterial agent may potentially contribute to differences in susceptibility to periodontitis and the course of the disease, the polymorphisms of the IL-1 encoding genes, as susceptibility markers for periodontitis, became a subject of special interest.

In paper by Kornman et al. (1997), on the basis of a study including 99 Caucasian non-smokers, described a strong correlation between severe chronic periodontitis and the presence of a specific IL-1 complex genotype. The genotype was characterized by the simultaneous presence of two gene polymorphisms, with allele 2 both in locus IL-1A^−889^ and in locus IL-1B^+3953^. In both cases, allele 2 was characterized by cytosine nucleotide replacement by thymine. Kornman et al. (1997) defined individuals with at least one altered IL-1A^−889^ allele and at least one altered IL-1B^+3953^ allele as having a positive genotype. In these individuals, the risk of severe chronic periodontitis was almost seven times higher than in those with a negative genotype, i.e., without simultaneous occurrence of at least one allele 2 in both loci. Among 71 subjects aged 40–60 years, the positive genotype was found in 78% of patients with severe periodontitis, in 26% of patients with moderate periodontitis, and in 16% of patients with mild periodontitis. A separate analysis of the incidence of allele 2 of IL-1A^−889^ and of IL-1B^+3953^ did not show an association of single genes with disease progression. In addition, there was no association between genotype (+) and the degree of disease severity in the 35 subjects who smoked. It was interpreted by the author as the major overcasting effect of nicotinism, being the main risk factor for periodontal disease, and indicating the necessity of excluding smokers from further studies (Kornman et al. 1997). The study was a cornerstone in understanding genetics as a predicting factor for the periodontal disease. It stood behind developing the commercially available genetic test, which in various brands is available until today (i.e., Ilustra by Interleukin Genetics Inc, USA; PGT by LabOral Diagnostics, Netherlands; Genotype PST by Hain Lifescience GmbH, Germany). The PST^®^ (Periodontal Susceptibility Test), also known in Europe as the PRT test, from the German Parodontitis Risiko Test, in its initial version used a sample of blood drawn from a finger, and now is using swabs from the buccal mucosa, which are sent to a laboratory for DNA analysis. Then, the IL-1A^−889^ and IL-1B^+3953^ alleles are identified by the polymerase chain reaction followed by reverse hybridization. In the case of simultaneous occurrence of at least one allele 2 in both loci, the result is referred to as PST^®^ positive, presumably indicating an increased risk of severe periodontitis. For 20 years, numerous polymorphisms of a single nucleotide have been linked with increased incidence or severe course of periodontal inflammation. A vast number of scientific centers spread worldwide conducted such studies which resulted in an increasing variety of the polymorphism description, and potentially might lead to confusion. Therefore, Human Genome Variation Society introduced the description nomenclature unifying the previously used one, last updated in 2016 (den Dunnen et al. 2016). Coherent to that is the description used by National Center for Biotechnology Information (NCBI). That description uses specific numbers to describe single nucleotide polymorphism (SNP), disregarding their location in the genome (Kitts and Sherry 2011). They are described as the reference-based single nucleotide polymorphism IDs (rsSNP IDs), and—despite voices that SNP approach may be obsolete in the era of genome-wide association studies (Hurgobin and Edwards 2017)—they are convenient in describing polymorphism influence on the phenotype.

## Methodology

The authors looked for rsIDs regarding IL-1 gene cluster through sources available in the NCBI database (https://www.ncbi.nlm.nih.gov/SNP/). The phrases used in the search were “IL1A” and “IL1B”. The search yielded 2919 and 2261 results, respectively. Those were filtered to the rsIDs cited in PubMed. That produced 27 and 24 results, respectively. Filtered rsIDs were manually evaluated regarding the papers in PubMed researching their influence on the periodontal inflammation (two and five results, respectively). A manual search of PubMed database was also performed to identify other potentially important studies. Special attention was paid to the credibility of the results: longitudinal studies and meta-analyses were pointed out. The results were presented with reference to the given rsIDs. Below the rsID, the following data were given: alteration of the genome (following the HGVS criteria) and global MAF (minor allele frequency—incidence of the minor allele in a default global population).

### IL1A Gene

***rs17561***

***Alteration: [G/T], Global MAF 0.218***

Yin et al. (2016) published a meta-analysis evaluating an association between the aforementioned SNPs and periodontitis. The search of the medical databases resulted in six case–control studies published between 2008 and 2014 fulfilling the selection criteria. According to the calculations, T allele of rs17561, both evaluated individually or as homozygous (TT) and heterozygous (CT) variant versus T allele non-carriers, showed a significant and positive correlation with greater periodontal risk (OR = 1.50 and 1.57, respectively) (Yin et al. 2016). rs17561 was also included in the examination of 280 Mexican individuals from San Luis Potosi state. The patients were divided into three equal groups, depending on their medical examination regarding periodontitis (PE) or rheumatoid arthritis (RA) (80 individuals in PE, RA and control groups) and a 40-person group of patients suffering from both PE and RA. The authors did not find any positive correlation between rs17561 and periodontitis, neither in Kruskal–Wallis test, nor in logistic regression analysis (Dominguez-Perez et al. 2017).

***rs1800587***

***Alteration: [C/T], Global MAF 0.279***

rs1800587 is also described as IL-1α[−889] and reported by Kornman et al. (1997)—together with IL-1β[+3953], further referred to as rs1143634—as a predictive factor for severe course of chronic periodontitis. Amongst other studies was a part of the meta-analysis performed by Karimbux et al. (2012). It included 27 studies published between 1997 and 2008. Nineteen of them reported a positive association with either periodontitis incidence or severity, either as alone SNP or in tandem with rs1143634 (Karimbux et al. 2012). The fact that four of those studies monitored patients for an extended period of time—in the United States 42 persons for 14 years (McGuire and Nunn 1999), in Australia 295 persons for 5 years (Cullinan et al. 2001), in Sweden 283 individuals for 10 years (Axelsson 2002), and in Switzerland 224 persons for 4 years (Persson et al. 2003) is worth noticing. According to the authors, all studies involving 200 or more subjects showed a statistically significant association between SNPs and periodontitis. Quantitative analyses of the eligible papers revealed an association of rs1800587, both alone and together with rs1143634, with clinical parameters of periodontitis. However, according to the authors, the observed and unexplained significant heterogeneity suggests treating the calculated odds ratio (OR) values with caution (Karimbux et al. 2012). In 2011, a case–control study on German patients with chronic and aggressive periodontitis was conducted to evaluate, whether any SNPs distribute differently in those patients while compared to healthy controls (Schulz et al. 2011). The study involved 159 periodontitis patients almost evenly distributed between chronic and aggressive periodontitis groups, and 89 periodontitis-free individuals. No association was found for the rs1800587 and chronic periodontitis, but there was, however, an association between the occurrence of this SNP—alone and in conjunction with the SNP of IL1B reported by Kornman et al. (1997) and pocket colonization with *Aggregatibacter actinomycetemcomitans*. Acknowledging the fact that this pathogen is reported to be responsible for aggressive onset and progress of the disease in adolescents and young adults, it might be concluded that the given genotype can be associated with severe forms of periodontal disease (Schulz et al. 2011). Another case–control study was performed among a Brazilian population of 113 subjects—healthy or with moderate and severe form of periodontitis—however, no association was found between rs1800587 and chronic periodontitis (Trevilatto et al. 2011). A genome-wide association study performed by Divaris et al. (2013) in United States showed no significant genome-wide signals for chronic periodontitis. According to the already mentioned case–control study by Dominguez-Perez et al. (2017) on Mexican population, rs1800587 was of borderline significance for association with periodontitis. Moreover, a significantly negative relationship (OR = 0.03) was found for the composite haplotype of rs1800587(C) and rs16944(A) (the latter one associated with IL1B gene) (Dominguez-Perez et al. 2017). Interestingly, a retrospective analysis performed by Jacobi-Gresser et al. (2013) showed an association of rs1800587 with prediction of titanium implant failure. Scientists from Oslo University did not observe an influence of rs1800587 on increased incidence of chronic periodontitis in 74 patients with vascular diseases (Armingohar et al. 2014). Zuccarello et al. (2014) also did not observe any correlations between that phenotype and occurrence of chronic periodontitis, which, however, correlated with parental history of the disease. Similar results were obtained by Japanese scientists (Tanaka et al. 2014). The geographical factor and heterogeneity of the studied populations might be a reason contributing to different results obtained by various scientific teams, since the study by Boukorrt et al. (2015) on Algerian population diagnosed with aggressive periodontitis (279 individuals) showed a significant correlation between the carriage rate of minor rs1800587 allele and the incidence of periodontal inflammation. da Silva et al. (2017) published a meta-analysis of 21 case–control studies published between 1998 and 2015, including some of the manuscripts described above (Armingohar et al. 2014; Boukorrt et al. 2015; Schulz et al. 2011; Trevilatto et al. 2011; Zuccarello et al. 2014). The meta-analysis included 3930 individuals of various races. The authors showed an association of rs180057 with periodontitis. T allele was associated with periodontitis cases, C allele with healthy cases (OR = 1.22 and 0.82, respectively). However, when evaluating the influence of ethnicity, that finding should be limited only to Caucasians—other ethnic groups did not show any linkage with rs1800587, possibly due to heterogeneity bias (da Silva et al. 2017).

### IL1B Gene

***rs1143627***

***Alteration: [C/T], Global MAF 0.472***

In 2017, scientists of Zhuhai People’s Hospital (Huang et al. 2017) published a meta-analysis of an association between periodontitis and rs1143627—a polymorphism commonly linked with cancer of the digestive (Li et al. 2017) and respiratory tract (Wang et al. 2017). The analysis comprised six case–control studies, published between 2008 and 2014. Those studies involved eight different patient groups (two studies included both chronic and aggressive periodontitis patients) and were conducted in India (two studies), Japan (two studies), Italy and Jordan (Huang et al. 2017). The overall comparison failed to show any significant correlation with periodontal disease susceptibility, however, a stratified analysis by ethnicity showed a correlation between TT homozygotes and increased risk of pocket depth occurrence in Jordanians. Egger’s test did not reveal any publication bias (Huang et al. 2017).

***rs16944***

***Alteration: [A/G], Global MAF 0.491***

Schulz et al. (2011) in the already described study on patients with aggressive periodontitis also evaluated rs16944. The authors did not observe any association between the mentioned polymorphism and both chronic and aggressive periodontitis. They noticed, however, a weak linkage disequilibrium with rs1143634, suggesting a non-random association between both genes (Schulz et al. 2011). In a study by Trevilatto et al. (2011), a correlation between rs16944 and pocket depth occurred in a group of Afro-Americans and mulattos. In 2015, joint scientific teams from United States, Chile and China published a study of four SNPs in four ethnic groups: Caucasians, Afro-Americans, Hispanics and Asians (Wu et al. 2015). The research revealed a statistically significant linkage between specific genotype patterns of the SNPs in IL1B promoter region (apart from rs16944 also rs1143623 and rs4848306) and moderate to severe periodontitis occurrence, also after adjusting the model for age, diabetes and smoking (Wu et al. 2015). Armingohar et al. (2014) did not observe any difference in distribution of rs16994 alleles among patients with periodontitis and vascular diseases. Moreover, in young Japanese women, heterozygotes were associated with reduced occurrence of periodontitis (Tanaka et al. 2014). Zeng et al. (2015) in a meta-analysis including 21 case–control studies did not find rs16944 to be associated with a risk of developing chronic periodontitis. Ribeiro et al. (2016) was investigating an interaction between selected IL1B polymorphisms and periodontal condition in Brazilian population. Hundred and forty-five individuals with healthy periodontium, chronic periodontitis or aggressive periodontitis were enrolled in the study. For rs16944, significant associations were not observed, neither for aggressive, nor for chronic periodontitis. Certain composite haplotypes, comprising three genes (apart from rs16944, also rs1143634 and rs2234663) presented strong associations with both periodontal diseases—depending on alleles’ composition (Ribeiro et al. 2016). In the earlier mentioned study by Dominguez-Perez et al. (2017) conducted on Mexican subjects, rs16944 was at the borderline of showing statistical significance with periodontal disease. Adenine containing haplotype (major allele) combined with major allele of rs17561 was negatively associated with periodontitis (Dominguez-Perez et al. 2017). Indian researchers showed no influence of rs16944 on the IL-1β levels in gingival crevicular fluid (Lavu et al. 2017). Huang et al. (2017) published a meta-analysis of 19 case–control studies (3900 healthy individual vs 2173 patients suffering from chronic periodontitis in total). The results suggested a non-significant linkage between rs16944 and periodontal inflammation, both in summarized analysis and in evaluation of subgroups (Huang et al. 2017).

***rs1143623***

***Alteration: [C/G], Global MAF 0.292***

***rs1143633***

***Alteration: [A/C/G], Global MAF 0.311***

rs1143623 and 1143633 were involved in the haplotype composition of three SNPs that Wu et al. (2015) showed to be in certain patterns linked with an increased risk of moderate to severe periodontitis. The OR for such an occurrence varied depending on the race and was ranging from 1.87 in Caucasians and Afro-Americans to 3.27 in Asians. The value obtained from the meta-analysis also supported the thesis of an association between the studied haplotype composition and periodontal inflammation (OR = 1.95, *p *< 0.001) (Wu et al. 2015).

***rs1143634***

***Alteration: [C/T], Global MAF 0.133***

rs1143634 is a polymorphism of IL-1B gene described by Kornman et al. (1997). Numerous following papers were considering composite genotype of two polymorphisms: rs1800587 (IL1A) and rs1143634 (IL1B). Studies have shown a significant variety of occurrence of positive genotype, depending on the race and geographical location. Most of those studies were carried out on Caucasians. The incidence of subjects with positive genotype (both second alleles present in the genotype) in larger epidemiological studies amounted to 34.3–38.9% of the population (Hodge et al. 2001; Meisel et al. 2004; Thomson et al. 2001). A meta-analysis performed in 2012 summarized studies regarding composite genotype of the two aforementioned SNPs, as well as rs1143634 alone (Karimbux et al. 2012). Global MAF resulting from an analysis of population cohort of 800 Caucasians equaled 0.22. It was higher than the value presented above, probably due to the fact that studies on different populations reveal lower frequency of minor allele (Gonzalez et al. 2003; Lopez et al. 2005; Um and Kim 2003; Walker et al. 2000). The meta-analysis showed that in larger samples (*n *> 100), IL1A, IL1B and composite gene variations correlated with the incidence and severity of periodontal inflammation. Basing on the ten studies eligible to be included in the analysis, OR for the association between rs1143634 and chronic periodontitis was 1.54 (1.51 for the composite genotype) (Karimbux et al. 2012). The occurrence of the minor allele of rs1143634 was higher in the group of patients with implant failure than in patients with successfully osteointegrated implant (Jacobi-Gresser et al. 2013). In a study by Schulz et al. (2011), composite genotype was associated with twice as high occurrence of *A. actinomycetemcomitans* in subgingival biofilm. However, a linkage between rs1143634 and severity of chronic periodontitis was not observed. Similarly, a genome-wide association study did not show a significant correlation between rs1143634 polymorphism and occurrence of moderate or severe chronic periodontitis (Divaris et al. 2013). rs1143634 was, unlike familiarity, not associated with periodontitis in Zuccarello’s study (Zuccarello et al. 2014). Also, in a study by Tanaka et al. (2014), neither of the studied polymorphisms was related with periodontal disease. Armingohar et al. (2014) in a study on Scandinavian patients scheduled for vascular surgery noticed no associations between rs1143634 and the state of periodontal tissues. Finally, a study conducted on 225 Colombians, either with periodontitis or healthy periodontal tissues, did not show a significant linkage between rs1143634 and periodontal inflammation (Isaza-Guzman et al. 2016). However, different papers yield opposite results. A study by Wu et al. (2015) in its discovery part revealed that specific pattern of four SNPs, including incidence of minor allele of rs1143634, was associated with higher risk of periodontitis (OR = 1.87). Very similar results were obtained during studies on two different races—Hispanics and Asians, with OR for the latter reaching the value of 3.27. The conducted meta-analysis of pooled populations confirmed that linkage (Wu et al. 2015). A study conducted on Algerian population showed a linkage between rs1143634 and aggressive periodontitis, with the OR value of 1.69 (Boukortt et al. 2015). The already cited study by Ribeiro et al. (2016) on 145 dwellers of Rio de Janeiro, healthy or diagnosed with aggressive or chronic periodontitis, included evaluation of the rs1143634 allele. The results showed increased incidence of the minor allele of rs1443634 among individuals with chronic periodontitis (OR = 2.84). The OR increased almost twofold if only smokers were evaluated, and was even higher in females (OR = 6.00). Also, polymorphism of rs1143634 combined with minor allele of gene encoding IL-1 receptor antagonist increased the risk of periodontal inflammation; the results were even higher if IL1A rs16944 gene was included (Ribeiro et al. 2016). Lavu et al. (2017) in their study did not notice any difference in IL-1β levels between carriers of major and minor rs1143634 allele, however, they observed that heterozygotes showed higher levels of IL-1β in gingival crevicular fluid. Also, a study on Mexican population (280 individuals) revealed an association of minor allele homozygotes (TT) with the occurrence of periodontitis, after adjustment for age, gender and nicotinism (Dominguez-Perez et al. 2017). Recently, a meta-analysis was published (da Silva et al. 2018), evaluating 54 case–control studies from the period between 1998 and 2016, including some of the studies discussed above (Armingohar et al. 2014; Boukorrt et al. 2015; Isaza-Guzman et al. 2016; Lavu et al. 2017; Schulz et al. 2011; Trevilatto et al. 2011; Zuccarello et al. 2014), and some of those included in the meta-analysis by Karimbux et al. (2012). That composed a summarized group of 4924 patients with chronic periodontitis and 4452 healthy controls. The results showed T allele to be associated with the study group, while C allele was associated with the healthy controls (OR = 1.35 and 0.74, respectively), which pointed towards a linkage between rs1143634 and the occurrence of periodontal inflammation. T allele was linked with elevated risk of periodontal disease in Caucasians, Asians, and in mixed population, but not in Africans (da Silva et al. 2018).

A summary of the meta-analyses on the described SNPs is presented in Table 1.

**Table 1:** Presentation of meta-analyses concerning selected single nucleotide polymorphisms (SNPs) of IL-1A and IL-1B genes

Gene	SNP	Meta-analyses (+)	Meta-analyses (−)
IL1A	rs17561	1 (189) Yin et al. (2016)	0
	rs1800587	2 (7097) Karimbux et al. (2012), da Silva et al. (2017)^a^	0
IL1B	rs1143627	0	1 (1078) Huang et al. (2017)
	rs16944	0	1 (6073) Zeng et al. (2015)
	rs1143623	0	0
	rs1143633	0	0
	rs1143634	2 (14,064) Karimbux et al. (2012),da Silva et al. (2018)^a^	0

Meta-analyses (±)—meta-analyses confirming or denying the association between the given SNP and periodontitis

Numbers in cells—number of studies (in brackets—total subjects evaluated)

^a^Both studies of da Silva et al. included some of studies evaluated by Karimbux et al

## Discussion

The paper by Kornman et al. (1997), though being a major step forward in understanding the impact of genome on outbreak, progress and outcome of periodontal inflammation did not evade criticism. One of the major issues is the fact that this is a cross-sectional study, and the correlation between the IL-1 genotype and the disease prevalence is calculated using OR. In the theory of probability, it simply calculates how the knowledge of one variable’s value influences the other variable’s value. Fitting that to the periodontitis–genome correlation, the OR only informs us how probable it is that in patients with severe periodontitis, we will discover positive genotype (and vice versa). Moreover, it does not relate to the timescale. Two variables may result from each other, but they also may be accidentally correlated (or other factors may be involved). A definite answer would be given by longitudinal studies. Those are lacking in the research on a linkage between IL-1 gene cluster and periodontitis. Only several were performed and included in one of the discussed meta-analyses (Karimbux et al. 2012). A deficiency of those in periodontal research may be easily explained. Technical difficulties in conducting such studies are obvious, but also is the necessity to account for numerous environmental factors influencing periodontal disease: smoking, obesity, coexisting diseases, diet, oral hygiene habits, dental caries, quality of dental restorations, etc. That raises another obstacle in explaining the role of genome in periodontal disease. Research conducted so far seems to prove that there is no single gene that could be used worldwide, in different ethnicities, to identify people prone to develop severe periodontitis. That leads to the obvious conclusion, that at least several genes are involved in periodontal disease susceptibility. It is possible that several dozens of genes can be associated with periodontitis. The more genes are involved, the bigger is the role of the other environmental factors mentioned above. Studies conducted in 1970’s showed that even a basic etiological factor—dental biofilm—in some cases is not sufficient to cause periodontal inflammation in gingivitis patients (Loe et al. 1978). Pathogenesis of periodontal disease is much more complex (Murakami et al. 2018), and all of the factors contributing to the disease must be taken into account. The OR for the studied SNPs increased when individuals with coexisting risk factors were selected (Karimbux et al. 2012).

As presented in Table 1, meta-analyses in most of the reported SNPs seem to show an association between the given polymorphism and the periodontal condition. Case–control studies, however, when examined separately, show inconsistent outcomes. This may result from the variety of ethnic groups examined, but seems to suggest that evaluation of a single SNP may have little or no prognostic/diagnostic value for clinicians. Evaluation of specific genetic patterns, consisting of two, three or more genes, may more accurately indicate individuals with greater risk of periodontal inflammation (Kornman et al. 1997; Wu et al. 2015).

## Conclusions

The presented scientific research brings inconsistent results. It is possible that ethnical heterogeneity may be the reason contributing to the diversity of the results. Also, geographical and racial distribution of the specific genes may make it difficult—or even impossible—to globally assess single nucleotide polymorphisms. New evaluation techniques, like standardization of the methodology allowing data meta-analysis, or genome-wide association studies, may shed more light on the linkage between genotype and the onset or progress of periodontitis. Meta-analyses show that at least two single nucleotide polymorphisms: rs1800587 and rs1143634, are associated with periodontal inflammation. Therefore, they can be regarded as candidate genes involved in further periodontitis risk assessment.
